# Comprehensive Analysis of Endogenous Volatile Compounds, Transcriptome, and Enzyme Activity Reveals *PmCAD1* Involved in Cinnamyl Alcohol Synthesis in *Prunus mume*

**DOI:** 10.3389/fpls.2022.820742

**Published:** 2022-02-18

**Authors:** Tengxun Zhang, Fei Bao, Aiqin Ding, Yongjuan Yang, Tangren Cheng, Jia Wang, Qixiang Zhang

**Affiliations:** Beijing Key Laboratory of Ornamental Plants Germplasm Innovation and Molecular Breeding, Beijing Laboratory of Urban and Rural Ecological Environment, Key Laboratory of Genetics and Breeding in Forest Trees and Ornamental Plants of Ministry of Education, National Engineering Research Center for Floriculture, School of Landscape Architecture, Beijing Forestry University, Beijing, China

**Keywords:** *Prunus mume*, floral volatiles, transcriptome, cinnamyl alcohol, cinnamyl alcohol dehydrogenase

## Abstract

Floral scent is an important economic and ornamental trait of *Prunus mume*. The floral volatiles from most cultivars of *P. mume* in composition exist significant differences. Cinnamyl alcohol was one of the main floral volatile compounds with distinct abundances in different cultivars, namely, ‘Zaohua Lve,’ ‘Zao Yudie,’ ‘Fenpi Gongfen,’ ‘Jiangsha Gongfen,’ and ‘Fenhong Zhusha.’ Based on the determination of endogenous volatiles of full-blooming flowers, vital enzyme activity and transcriptomes were comprehensively analyzed to screen the key potential genes involved in cinnamyl alcohol synthesis. Transcriptome combining with enzyme activity level analysis suggested that the expression levels of three *PmCADs* were highly correlated with the cinnamyl alcohol dehydrogenase (CAD) enzyme activities in six cultivars. Furthermore, phylogenetic tree and transcriptome analysis suggested that PmCAD1 and PmCAD2 might contribute to the cinnamyl alcohol synthesis. Relative expression analyses and enzyme activity assays showed that PmCAD1 played an important role in cinnamyl alcohol biosynthesis *in vitro*. Overall, this research lays a theoretical foundation for clarifying comprehensively the molecular biosynthesis mechanism of floral volatiles in *P. mume*.

## Introduction

*Prunus mume* is a traditional woody flower in China, with high aesthetic and ornamental value. Its fragrance is an important feature that distinguishes it from other *Prunus* genus in the *Rosaceae* family (Hao et al., 2014). In addition, the volatile organic compounds are the main components of plant essential oils, which provide important resources for the development of plum perfume and are used in various perfumes, soaps, and cosmetics (Muhlemann et al., 2014).

It was reported that phenylpropanoids/benzenoids, including benzyl acetate, eugenol, benzyl alcohol, cinnamyl alcohol, cinnamyl acetate, and benzyl benzoate, were the main components contributing to the floral volatiles from *P. mume*. Among the exogenous volatiles of ‘Fenpi Gongfen,’ ‘Jiangsha Gongfen,’ and ‘Fenhong Zhusha,’ cinnamyl alcohol was a unique compound that distinguished between these three cultivars and other five cultivars, such as ‘Zaohua Lve’ and ‘Zao Yudie’ (Zhang T.X. et al., 2020). Cinnamyl alcohol was derived from phenylpropanoid/benzenoid biosynthesis. Previous studies have reported that a series of enzymes in this pathway involved in the synthesis of cinnamyl alcohol in plants, such as phenylalanine ammonia-lyase (PAL) (MacDonald and D’Cunha, 2007), cinnamoyl-CoA reductase (CCR) (Hsu et al., 2012), and cinnamyl alcohol dehydrogenase (CAD).

Cinnamyl alcohol dehydrogenase belonged to the medium-chain dehydrogenase/reductase (MDR) family and could directly catalyze cinnamaldehyde (coniferaldehyde, sinapaldehyde, and coumaraldehyde) to produce the corresponding cinnamyl alcohols. *CAD* genes were also a multigene family in plants, and CADs in different families had different affinities for the same substrate. It has been reported in various plants, such as *Arabidopsis thaliana* (Sibout et al., 2005), *Nicotiana tabacum* (Knight et al., 1992), *Oryza sativa* (Tobias and Chow, 2005), *Artemisia annua* (Ma et al., 2018), and *Sorghum bicolor* (Jun et al., 2017). The role of CAD in the synthesis of floral compounds was thought to catalyze the cinnamaldehyde to form cinnamyl alcohol. AaCAD from *A. annua* could catalyze geranial, cinnamaldehyde, sinapaldehyde, coniferaldehyde, and artemisinic aldehyde to produce geraniol, cinnamyl alcohol, sinapyl alcohol, coniferyl alcohol, and artemisinol, respectively (Li et al., 2012). Except in plants, ScAdh6p in the CAD family from *Saccharomyces cerevisiae* could catalyze cinnamaldehyde to cinnamyl alcohol with high catalytic activity, while it had low catalytic activity for sinapaldehyde and coniferaldehyde (Valencia et al., 2004). Therefore, the key CAD enzyme catalyzing the synthesis of cinnamyl alcohol in *P. mume* was worth studying.

To elucidate cinnamyl alcohol biosynthesis molecular mechanism clearly, transcriptome combining with metabolome analysis was an effective method, which has been applied in many plants, such as *Chimonanthus praecox* (Shang et al., 2020), *Rosa chinensis* (Raymond et al., 2018), *Nymphaea colorata* (Zhang L.S. et al., 2020), *Osmanthus fragrance* (Yang et al., 2018), and *Paeonia suffruticosa* (Zhang et al., 2021). Although the high-throughput RNA sequencing (RNA-Seq) regarding floral scent metabolism in *P. mume* has been reported (Zhao et al., 2017; Bao et al., 2020), few studies focused on the differences of floral volatile compounds from different *P. mume* cultivars.

Based on the above, we measured the endogenous VOCs to further analyze the differences in floral composition from six cultivars of *P. mume*. Then, transcriptome sequencing combined with the analysis of key enzyme activities was performed to screen the crucial enzyme genes related to the synthesis of floral volatiles. Finally, the candidate genes were cloned and characterized. This research provides a reference for the development of related derivative products of flower fragrance from *P. mume*. At the same time, analyzing the fragrance metabolism mechanism provides a theoretical basis for the fragrance breeding of *P. mume*.

## Materials and Methods

### Plant Material

Flowers of six *P. mume* cultivars were selected as plant materials, including *P. mume* ‘Zaohua Lve’ (Zah), *P. mume* ‘Zao Yudie’ (Zao), *P. mume* ‘Fenpi Gongfen’ (Fenp), *P. mume* ‘Jiangsha Gongfen’ (Jia), *P. mume* ‘Fenhong Zhusha’ (Fenh), and *P. mume* ‘Wuyuyu’ (Wuy). The characters of flowers could be seen in the study by Zhang T.X. et al. (2020). To analyze the endogenous volatile compounds and transcriptome, the whole flowers of six cultivars at the blossom stage were collected in liquid nitrogen before being stored at the –80°C refrigerator.

The entire flowers of ‘Fenh’ at different flowering stages were used to analyze the emission rhythm of cinnamyl alcohol and cinnamyl acetate. Four flowering stages of flower included the following: budding stage (BS): flower did not open at all; initial flowering stage (IFS): flower was slightly opened; full-blooming stage (FS): flower was fully opened; wilting stage (WS): petals started to fall.

### Extraction and Gas Chromatography-Mass Spectrometry Analysis of Volatile Compounds in Flower

The whole flowers were ground in liquid nitrogen, and 0.2 g of powders were extracted with 1 ml ethyl acetate containing 10 ng of benzyl propionate as internal standard. Following continuous shaking on a vortex mixer for 15 min, samples were centrifuged at 13,000 rpm for 10 min at room temperature and then transferred the supernatant extraction into a new 2 ml centrifugal tube. The extraction was dried using anhydrous sodium sulfate and collected. Each sample was performed on three biological replicates.

The endogenous extracts were determined at the Testing and Analysis Center of Beijing Forestry University. The instrument used was GPC-GC (Shimadzu, Kyoto, Japan) equipped with chromatographic column Rtx-5MS (30 m × 0.25 mm × 0.25 μm, Shimadzu, Kyoto, Japan). Notably, 1 μl of each sample was injected through the automatic sampler for analysis. The oven and injection temperature were 40 and 280°C, respectively. The carrier gas was helium in the split mode (split ratio: 20), and the column flow rate was 1 ml/min. The total program time was 50.5 min. The detailed steps were as follows: hold at 60°C for 2 min; increased to 150°C at a rate of 5°C/min; then increased to 280°C at a rate of 20°C/min; and hold for 20 min. The interface temperature of the mass spectrometer was 280°C, and the mass scanning range was 50–400 *m*/*z*.

The emission amounts of cinnamyl alcohol and cinnamyl acetate at different flowering stages of *P. mume* ‘Fenh’ were detected using headspace solid-phase micro-extraction combined with gas chromatography-mass spectrometry (GC-MS) (HS-GC-MS). After being weighed, the whole flower at every flowering stage was placed into a 25 ml injection vial and then held for 10 min. Extraction fiber coated with 50/30 μm divinylbenzene/carboxen/polydimenthylsiloxane (DVB/CAR/PDMS) was used to collect the volatiles for 30 min at 30°C. Three experimental replicates were conducted.

### Qualitative and Quantitative Analyses of Endogenous Extracts

The compounds were identified by comparing them with authentic standard samples. The detailed method could refer to Zhang T.X. et al. (2020). Notably, 1 ml of standard mixture dissolved in ethyl acetate contained 10 mg/L of each standard compound (benzaldehyde, benzyl alcohol, benzyl acetate, cinnamyl alcohol, cinnamyl acetate, eugenol, benzyl benzoate, and benzyl propionate). The intracellular volatile amounts were calculated from the following equation:


$$M_{\text{sx}}(\text{μg}/\text{g fresh weight})=A_{\text{sx}}/\left(A_{\text{ix}}/M_{\text{ix}}\right)/\left(A_{\text{s}}/A_{\text{i}}\right)/m$$

where *M*_*sx*_ is the amount of a compound measured in the sample, *A*_*sx*_ is the peak area of a compound in the sample, *A*_*ix*_ is the peak area of a compound in the standard mixed solution, *M*_*ix*_ is the amount of a compound in the standard mixed solution, *A*_*s*_ is the peak area of the internal standard in the sample, *A*_*i*_ is the peak area of the internal standard in the standard mixed solution, and *m* is the mass of the flower powder (g).

### RNA Extraction and Transcriptome Sequencing

The whole flowers of six cultivars (the same as the “Plant Material” section) at the full-blooming stage were used to construct cDNA libraries. The total RNA from the whole flowers was extracted according to the manual introduction of OMEGA Plant Total RNA Extraction Kit (R6827-01) with slight changes. The amount, purity, and integrity of total RNA were checked. The total RNA that met the conditions (RIN value > 7.0, concentration > 50 ng/μl, OD260/280 > 1.8, and extraction volume > 1 μg) was selected for further cDNA synthesis. Eighteen libraries were constructed using Illumina NovaSeq™ 6,000 (LC Biotechnology Co., Ltd., Hangzhou, China) to perform pair-end sequencing with PE150 mode.

### Sequence Assembly, Annotation, and Functional Classification

The clean reads were obtained in the format of fastq.gz after removing the low-quality and repeated sequences. The clean reads were compared with the genome of *P. mume* using HISAT (Kim et al., 2015). In addition, they were assembled using StringTie (Pertea et al., 2015). The annotation results were compared with the reference genome using gffcompare software.

### Gene Expression Levels and Differential Expressed Gene Analyses

R language software package (ballgown) was used to analyze the expression of each transcript, and the Fragments Per Kilobase of transcript per Million fragments mapped (FPKM) value was used to quantify the expression. The differences among samples were performed using edgeR (Robinson et al., 2010). In addition, the genes with a *p*-value < 0.05 were defined as differentially expressed genes (DEGs) among six cultivars.

### Quantitative Real-Time PCR

To validate the expression levels of DEGs, 1.0 μg of total RNA of each sample of *P. mume* cultivars were used as a template to synthesize cDNA following the recommendation of PrimeScript™ RT reagent Kit with gDNA Eraser (RR047A, TaKaRa, Dalian, China) kit of manufacturers. The TB Green™ Premix Ex Taq™ II (RR820A, TaKaRa, Dalian, China) was chosen to detect the relative expression level of the gene. Based on the CDS sequence of DEGs in the genome datasets, primers were designed using the online tool IDT.^1^ The primer sequences are shown in Supplementary Table 1. Among these, the *PmPP2A* gene was selected as the internal reference gene (Bao et al., 2020). The 10 μl reaction system included 2 μl cDNA (diluted 100 times), 0.2 μl each of forward and reverse primer, 5 μl TB Green II Premix Ex Taq enzyme and 2.6 μl ddH_2_O. The quantitative real-time PCR (qRT-PCR) was carried out on the PikoReal 96 real-time PCR system (Thermo Fisher) following the procedure: 95°C for 30 s, 40 cycles of 95°C for 10 s, and 60°C for 30 s. Each sample was repeated three times, and the relative expression level was calculated using the 2^–△△*Ct*^ method.

The expression levels of PmCAD1 and *PmCAD2* in the leaves of *Nicotiana benthamiana* were examined as above. *NtEF*α*1* was used as the internal reference gene. In addition, primer sequences are shown in Supplementary Table 3.

### Enzyme Activity Determination of PmCADs in *Prunus mume*

The extraction method of crude protein of flowers is referred to Bao et al. (2019). To measure the activity of CAD in each cultivar, 2 ml of reaction buffer containing 150 μg crude protein, 20 mM 2-(N-Morpholino) ethanesulfonic acid (MES) (pH = 6.0), 200 μM NADPH, and 1 mM cinnamaldehyde and ddH_2_O were incubated in a metal bath at 30°C for 1 h. After extraction with 500 μl of ethyl acetate containing 5 ng of benzyl propionate as the internal standard, the reaction product was detected by GC-MS, and the procedure of which was the same as mentioned above.

### Phylogenetic Analysis, Gene Clone, and Vector Construction

After multiple sequence alignment using Clustal X software, the phylogenetic tree was constructed using MEGA10 with the neighbor-joining method and 1,000 bootstrap replicates. In the phylogenetic tree, the accession numbers of CAD sequences are shown in Supplementary Table 2.

The open reading frame (ORF) of *PmCADs* was obtained using the genome data of *P. mume*, and primers were designed. cDNA of each cultivar obtained in the “Quantitative Real-Time PCR” section was as templates for PCR amplification. Sequences were amplified with KOD DNA polymerase (TOYOBO, Japan), and the procedure was performed as follows: 94°C for 2 min, 30 cycles of 94°C for 15 s, 55°C for 30 s, 68°C for 1 min, and 68°C for 5 min. The ORF of *PmCADs* was cloned into the *pSuper1300-GFP* vector for the transformation of tobacco leaves. Restriction endonucleases, namely, *Xba*I and *Kpn*I, linearized this vector. Then, the In-Fusion ^®^ HD Cloning Kit (Clontech, CA, United States) was used to construct the recombinant plasmid. Similarly, the prokaryotic expression vector was constructed. The ORF of *PmCAD1* was cloned into the *pET-30a* vector *via Nde*I and *Xho*I sites. Primers are shown in Supplementary Table 3.

### Expression in Leaves of Tobacco and Characterization of *PmCADs*

*PmCADs* were overexpressed in leaves of *N. benthamiana* to obtain the mass of PmCAD protein. The *Agrobacterium tumefaciens* with *pSuper1300-PmCADs-GFP* vector in MES buffer was injected into the leaves. The leaves were collected in liquid nitrogen after being cultured 56 h. The total RNA of leaves of *N. benthamiana* was extracted to detect the expression levels of *PmCADs*. In addition, crude protein of leaves was extracted. The system of PmCADs enzyme assay was the same as the “Enzyme Activity Determination of PmCADs in *P. mume*” section. After 1 h of reaction, the absorbance was measured at 340 nm. The reaction products were detected using GC-MS. In addition, this method was the same as the PmCADs enzyme assay in *P. mume*. The amount of 1 nM NADPH consumed per mg of crude protein per minute was an enzyme activity unit (U/mg).

### Heterologous Expression of PmCAD1 Protein in *Escherichia coli*, Protein Purification, and Enzyme Assay *in vitro*

For inducing the recombinant pET-30a-PmCAD1 protein, the *Escherichia coli* strain ArcticExpress cells containing recombinant plasmid were cultured in 300 ml of Luria-Bertani (LB) medium containing 50 μg/ml kanamycin with shaking at 37°C for 4 h. When OD600 value of suspension culture reached approximately 0.6, 0.2 mM isopropyl β-thiogalactopyranoside (IPTG) was added. Then, the suspension culture was brought to 15°C overnight with shaking. The protein was purified using the Ni-IDA-Sepharose Cl-6B column according to the protocol. The purified sample was identified using SDS-PAGE analysis. Bradford protein assay with BSA as a standard was used to detect the protein concentration.

For enzyme kinetics analysis, cinnamaldehyde of five different concentrations (i.e., 20, 40, 60, 100, and 200 μM) was used as substrate. Notably, 200 μl of reaction volume contained 2 μg purified protein, 2 mM dithiothreitol, 300 μM NADPH, 50 mM phosphate buffer (pH = 6), and cinnamaldehyde. All reactions were carried out in 96-well microplates. The absorbance was measured at 340 nm with a microplate reader (Infinite M200pro, Tecan, Switzerland), and the procedure of which was set at 30°C and recorded values every 2 min for 10 min. Each experiment was performed with three replicates. *K*_*m*_ and *V*_*max*_ were calculated using the Michaelis-Menten equation using GraphPad Prism 8 software.

### Data Analysis

The correlation analysis between gene expression level and enzyme activity was conducted using the Omicstudio tool^2^ with the Pearson calculation method. Microsoft Excel 2019 was used to count experimental data, and the significance of which was analyzed using SPSS 23.0 (Chicago, United States) with one-way ANOVA.

## Results

### The Intracellular Pool Sizes of Volatile Compounds in Flower

The floral volatiles in the intracellular pools of six cultivars were extracted using ethyl acetate and detected with GC-MS. In this study, we analyzed the amounts of the following seven compounds: Benzaldehyde, benzyl alcohol, benzyl acetate, eugenol, cinnamyl alcohol, cinnamyl acetate, and benzyl benzoate. The absolute content of each compound is shown in Table 1. The benzaldehyde amount was higher than other compounds in each cultivar. In addition, its amount was the highest in ‘Zah’ and the lowest in ‘Jia.’ There was no significant difference in its content between ‘Zao’ and ‘Wuy.’ Benzyl alcohol content was the highest in ‘Jia,’ but it was not detected in ‘Wuy.’ The amounts of benzyl acetate detected in the tested four cultivars were relatively low. Benzyl benzoate was only detected in the endogenous extractions from ‘Zah.’ Only two compounds, namely, benzaldehyde and eugenol, were detected in ‘Wuy.’ The eugenol content in the endogenous volatiles of ‘Wuy’ was higher than that of other cultivars. Cinnamyl alcohol and cinnamyl acetate were the two main compounds that distinguished between ‘Fenp,’ ‘Jia,’ ‘Fenh,’ and the other three cultivars. Cinnamyl alcohol was detected in three cultivars, namely, ‘Fenp,’ ‘Jia,’ and ‘Fenh,’ among which the amount in ‘Fenp’ was far more than those in the other two cultivars. Cinnamyl acetate was only detected in ‘Fenp’ and ‘Fenh,’ and the content in ‘Fenp’ was higher than that in ‘Fenh.’

**TABLE 1:** Endogenous amount of main floral scent compounds in the six *Prunus mume* cultivars.

Compounds	Endogenous amount [mean ± SD (μg/g fresh weight)]					
	‘Zah’	‘Zao’	‘Fenp’	‘Jia’	‘Fenh’	‘Wuy’
Benzaldehyde	4662.8 ± 127.3	2491.4 ± 154.2	2769.3 ± 27.5	1445.4 ± 46.8	1675.0 ± 58.9	2360.7 ± 89
Benzyl alcohol	224.8 ± 48.7	63.5 ± 3.2	287.9 ± 37.2	393.0 ± 11.7	135.0 ± 12.5	–
Benzyl acetate	92.9 ± 12.2	92.4 ± 3.1	12.2 ± 0.5	26.4 ± 3.6	10.8 ± 0.7	–
Cinnamyl alcohol	–*^a^*	–	1344.7 ± 63.2	455.1 ± 34	311.3 ± 41.6	–
Eugenol	47.3 ± 3.8	65.7 ± 3.5	51.9 ± 2.4	35.8 ± 2	40.2 ± 2.7	90.9 ± 1.1
Cinnamyl acetate	–	–	76.6 ± 2.8	–	36.0 ± 2.9	–
Benzyl benzoate	199.8 ± 6.9	–	–	–	–	–

*^a^Not detected. The value is the mean of three biological replicates, and SD is also performed with three replicates.*

### The Emission Amount of Cinnamyl Alcohol and Cinnamyl Acetate at Different Flowering Stages of *Prunus mume* ‘Fenh’

**FIGURE 1:**
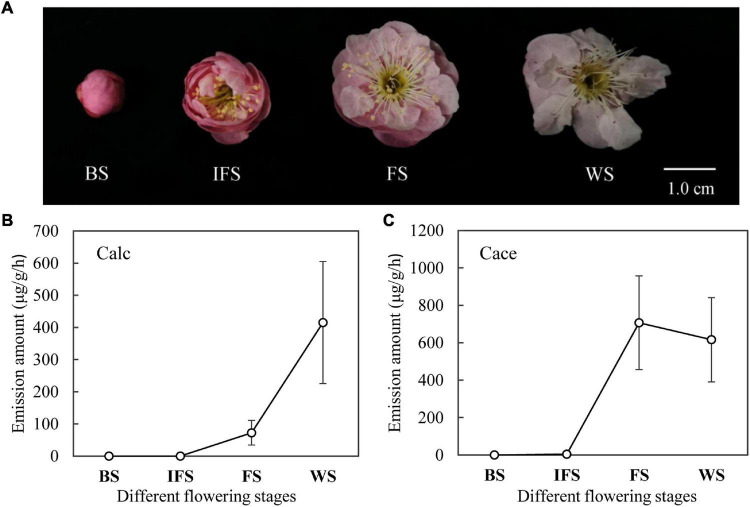
The release of cinnamyl alcohol and cinnamyl acetate at different flowering stages of *Prunus mume* ‘Fenh.’ **(A)** Flowers of *P. mume* ‘Fenhong Zhusha’ at different flowering stages: BS, budding stage; IFS, initial flowering stage; FS, full-blooming stage; and WS, wilting stage. **(B,C)** Dynamic changes of cinnamyl alcohol (Calc) and cinnamyl acetate (Cace) released at different flowering stages of *P. mume*, respectively.

To explore the temporal rhythm of emission, volatile amounts of cinnamyl alcohol and cinnamyl acetate at different flowering stages of ‘Fenh’ were detected using HS-GC-MS (Figure 1). The results showed that the release of cinnamyl alcohol and cinnamyl acetate at different flowering stages presented a dynamic change. The release amount of cinnamyl alcohol increased from the budding stage to the final flowering stage (Figure 1B). A similar emission pattern of cinnamyl acetate was found, and the emission peak appeared at the full-blooming stage. Then, the emission amount declined slightly at the wilting stage, while the change of its amount was not significant (Figure 1C).

### Transcriptome Analysis of Flowers From Six Cultivars

**FIGURE 2:**
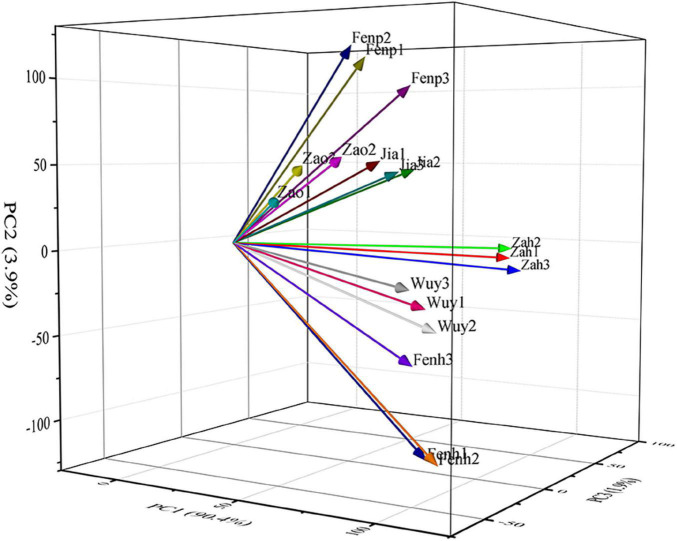
Principal component analysis of the samples.

To identify the DEGs related to the floral scent metabolism in flowers of these six cultivars, 18 cDNA libraries that were built with flowers at the full blooming stage were sequenced. After quality control of raw data, valid data were generated. As shown in Supplementary Table 4, the data quality was high, 40,900,532–52,060,556 clean reads were obtained from each library, and the valid ratio ranged from 96.29 to 98.88%. The Q20 and Q30 of 18 samples were greater than 99 and 97%, respectively. The GC content was approximately 45–46%. Mapping the clean reads to the reference genome of *P. mume*, the percentage of mapped reads was 89.47–93.86 (Supplementary Table 5). The correlation analysis showed that the correlation coefficient of three replicate samples of each cultivar was higher (> 0.86) (Supplementary Figure 1). In addition, the principal component analysis showed that the variances of PC1, PC2, and PC3 were 90.4, 3.9, and 1.9%, respectively, indicating there were variances among these samples (Figure 2).

### Expression Analysis of Differentially Expressed Genes That Involved in Phenylpropanoid/Benzenoid Biosynthesis

**FIGURE 3:**
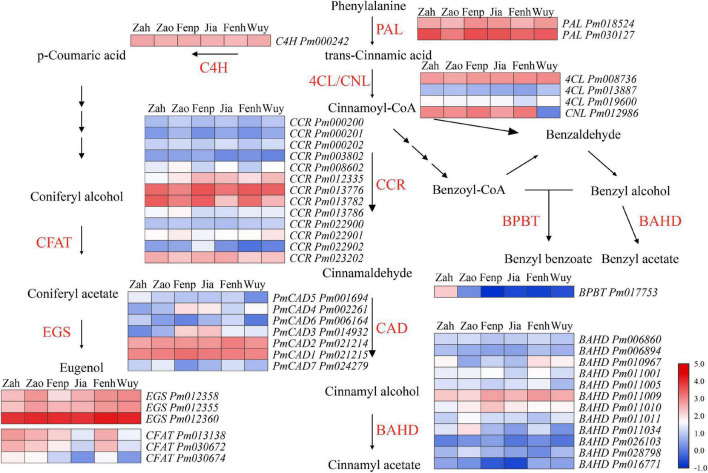
Expression analysis of genes in phenylpropanoid/benzenoid pathway of flowers at full-blooming stage of six cultivars. Red and blue represent a high and low FPKM value, respectively. The FPKM values were log10 transformed.

It was found that the floral volatile compounds from *P. mume* cultivars were mainly synthesized by the phenylpropanoid/benzenoid pathway. To explain the molecular metabolism of floral volatiles from *P. mume*, the differentially expressed structural genes involved in this pathway were analyzed. According to the KEGG and GO annotation, 46 DEGs that might involve in floral phenylpropanoid/benzenoid synthesis in *P. mume* were screened. The FPKM value and expression pattern of each gene are shown in Supplementary Table 6 and Figure 3, respectively. PAL was considered to be the first and the rate-limiting enzyme in the phenylpropanoid/benzenoid pathway (Dixon and Paiva, 1995; MacDonald and D’Cunha, 2007), which catalyzed the production of *t*-cinnamic acid from phenylalanine. Two differentially expressed *PmPALs* genes (i.e., *Pm030127* and *Pm018524*) were screened, and the FPKM value of *Pm030127* was much higher than that of *Pm018524*. It was reported that, in flowering plants, the synthesis of t-cinnamic acid from cinnamoyl-CoA was catalyzed by 4-coumarate: CoA ligase (4CL) in the cytoplasm. Three *Pm4CLs* were identified, and the expression level of *Pm008736* was much higher than the other two genes in every cultivar. CCR catalyzed cinnamoyl-CoA thioesters to generate corresponding cinnamaldehyde. The expression levels of thirteen *PmCCRs* showed differential patterns, among which four *PmCCRs* were highly expressed. The aldehydes were reduced to alcohols under the action of the CAD family, and seven *PmCADs* exhibited differential expression. A total of 16 *PmBAHD* genes have been identified, including the *PmBPBT* gene (*Pm017753*) involved in benzyl benzoate synthesis and three *PmCFATs* related to the synthesis of coniferyl acetate (Zhang et al., 2019). Additionally, it has been reported that *Pm011009* (*PmBEAT36*) and *Pm011010* (*PmBEAT37*) played significant roles in the synthesis of benzyl acetate (Bao et al., 2019). EGS catalyzed the synthesis of eugenol from coniferyl acetate, and three *PmEGSs* with high FPKM values were identified.

### Quantitative Real-Time PCR Verification of Gene Expression

To verify the reliability of the transcriptome data, we selected 17 genes, including structural genes and transcription factors in the phenylpropanoid/benzenoid biosynthesis pathway for qRT-PCR verification. The results showed that expression patterns of 17 genes were basically consistent with the RNA-Seq data (Supplementary Figure 2), indicating that the transcriptome data had high reliability and credibility.

### Correlation Analysis Between Enzyme Activity and Gene Expression Levels of *PmCADs*

**FIGURE 4:**
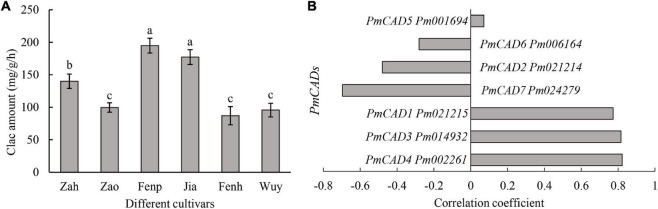
Screening of key *PmCADs via* enzyme activity and correlation analyses. **(A)** Enzyme activities that cinnamaldehyde to product cinnamyl alcohol of different *P. mume* cultivars. Calc: cinnamyl alcohol. The value represents the mean of three samples, and SD is also performed with three replicates. The lowercase letters represent the significant differences (*p* < 0.05). **(B)** Correlation analysis between enzyme activity and gene expression of *PmCADs* in six *P. mume* cultivars.

Cinnamyl alcohol was the important intermediate metabolite in the synthesis of cinnamyl acetate. Moreover, cinnamyl alcohol was one of the main compounds that distinguished between ‘Fenp,’ ‘Jia,’ ‘Fenh,’ and other three cultivars. CAD catalyzed cinnamaldehyde into cinnamyl alcohol. Thus, CAD activities were compared among six cultivars. As shown in Figure 4A, CAD activities in ‘Fenp’ and ‘Jia’ were obviously higher than that in the other four cultivars, followed by that in ‘Zah.’ There was no significant difference in CAD activities among ‘Zao,’ ‘Fenh,’ and ‘Wuy.’

To screen the potential *PmCAD* genes functioned on cinnamyl alcohol synthesis, correlation analysis between enzyme activity values and gene expression levels of *PmCADs* was performed. The results showed that the expression levels of three *PmCADs* (i.e., *PmCAD1*, *PmCAD3*, and *PmCAD4*) were highly positively correlated with cinnamyl alcohol yield in the enzyme reaction (Figure 4B).

### Identification and Sequence Analyses of *PmCADs*

**FIGURE 5:**
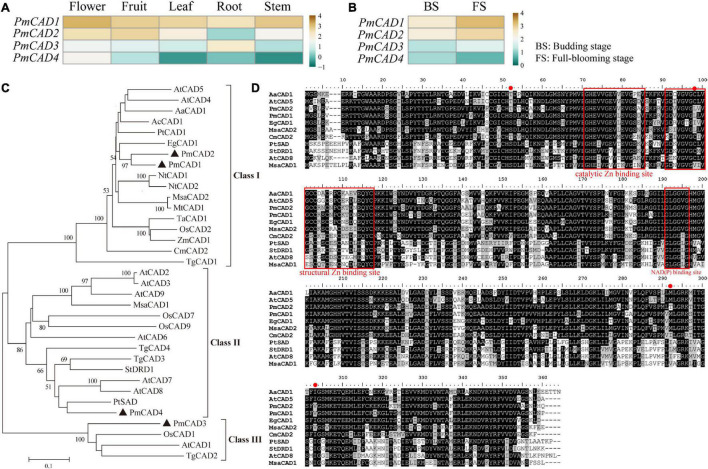
Expression patterns and sequence analyses of candidate *PmCADs*. **(A)** Expression patterns in five tissues (SRP014885); **(B)** expression patterns during the budding stage and full-blooming stage in transcriptome data sets (PRJCA000274). The RPKM values were log10 transformed. **(C)** Phylogenetic tree analysis of CAD amino acid sequence in *P. mume* and other species. **(D)** Multiple sequence alignment analysis of CADs in *P. mume* and other species. Their conserved domains were boxed and marked in the red box. GenBank annotation numbers are listed in Supplementary Table 2.

Due to the high sequence similarity (more than 85%) with *PmCAD1*, *PmCAD2* was also selected as one of the candidate genes for further study. Genbank annotation numbers of them are listed in Supplementary Table 2. Floral scent was emitted from the flower in *P. mume*, and the release of floral volatiles had temporal and spatial specificity (Zhang et al., 2019). To further explore the relationship between PmCADs and the synthesis of cinnamyl alcohol, the expression pattern of *PmCADs* in different tissues and at different flowering stages of *P. mume* was analyzed based on the RNA-seq data reported. The results showed that *PmCADs* were expressed in all five different tissues. *PmCAD1* and *PmCAD2* expressed highly in flower and fruit, while the expression level of *PmCAD3* in each tissue was lower than that of *PmCAD1* and *PmCAD2*, and it expressed more in the root. *PmCAD4* expressed most in flowers compared with other tissues (Figure 5A). The expression levels of *PmCAD1-3* genes increased from the budding stage to the full-blooming stage, which was consistent with the floral release pattern, while the expression pattern of *PmCAD4* was the opposite (Figure 5B). Therefore, it was suggested that *PmCAD1* and *PmCAD2* might play major roles in cinnamyl alcohol synthesis.

To study the evolutionary relationship between PmCADs and CADs from other species and further speculate on their roles in cinnamyl alcohol synthesis, a phylogenetic tree was constructed. As shown in Figure 5C, the phylogenetic tree divided CADs into 3 groups. PmCAD1 and PmCAD2 closed to EgCAD from *Eucalyptus globulus* were clustered into Class I, in which basically, every CAD had been characterized. PmCAD4 was clustered in Class II, and PmCAD3 was grouped into Class III, in which the function of CAD needed to be explored.

To further identify the PmCAD function on cinnamyl alcohol, the gene sequences of *PmCAD1* and *PmCAD2* were obtained from the six cultivars. Multiple sequence alignment of PmCAD1 and PmCAD2 from six cultivars are shown in Supplementary Figures 3, 4, respectively. The amino acid sequence identity of PmCAD1 from six cultivars was 99.81% and PmCAD2 99.91%. Due to the high similarity, the encoded sequences of *PmCAD1* and *PmCAD2* from ‘Fenh’ were used for further analysis. The ORFs of *PmCAD1* and *PmCAD2* were 1,071 bp and 1,074 bp in length, and the encoded amino acids were 356 and 357 aa, respectively. Several sequences of CADs from other species that have been reported to catalyze cinnamaldehyde to form cinnamyl alcohol were selected for multiple sequence comparisons. As shown in Figure 5D, the amino acid sequences encoded by PmCAD1 and PmCAD2 contained three conserved domains, namely, the catalytic Zn^2+^ binding site GHEX_2_GX_5_GX_2_V, the structural Zn^2+^ binding site GDX_10_CX_2_CX_2_CX_7_C, and NAD(P) binding site GLGGXG.

### The Relative Expression of PmCAD1 and PmCAD2 at Different Flowering Stages of *Prunus mume* ‘Fenh’

**FIGURE 6:**
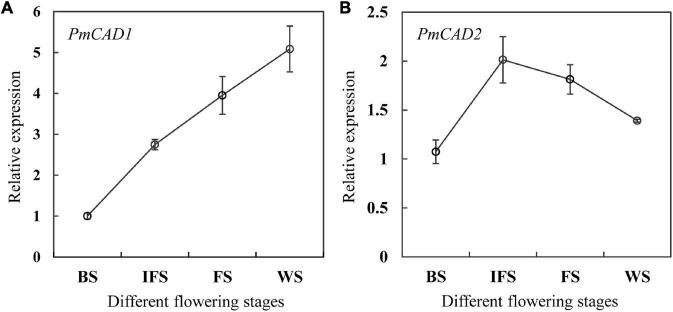
The relative expression levels of *PmCAD1-2* at different flowering stages of *P. mume* ‘Fenh.’ **(A,B)** Relative expression of *PmCAD1* and *PmCAD2* at different flowering stages of *P. mume*, respectively.

To further determine the role of PmCADs in the synthesis of cinnamyl alcohol, the relative expression levels of *PmCADs* at different flowering stages of *P. mume* ‘Fenh’ were performed using qRT-PCR. The expression level of *PmCAD1* gradually increased from the budding stage to the final flowering stage, which was consistent with the release of cinnamyl alcohol (Figure 6A). In addition, the expression level of *PmCAD2* raised from the budding stage to the initial opening stage, which reached the maximum, and then showed a gradual decrease (Figure 6B). The expression trend of *PmCAD1* instead of *PmCAD2* was the same as the release of cinnamyl alcohol, suggesting that PmCAD1 might play an important role in the synthesis of cinnamyl alcohol.

### Enzyme Activity Analysis of *PmCADs* in Tobacco Leaves

**FIGURE 7:**
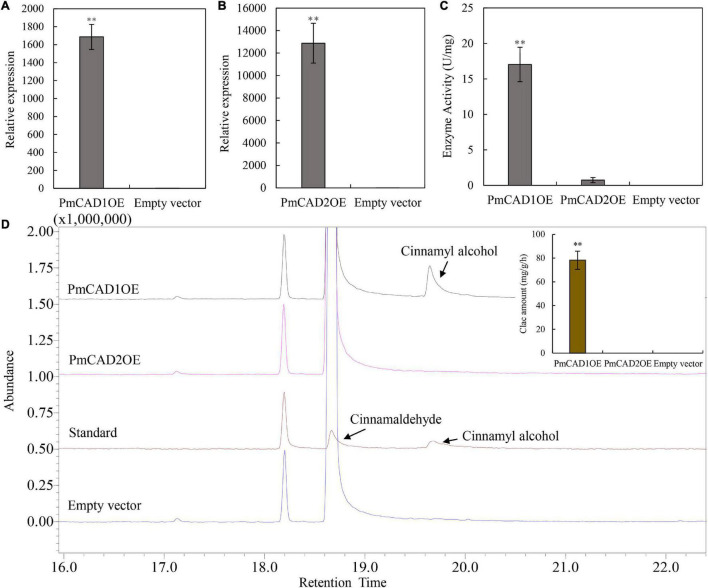
Enzyme activity analyses of PmCAD1 and PmCAD2. **(A,B)** Expression level of genes in tobacco leaves overexpressing *PmCAD1* and *PmCAD2*, respectively. **(C)** Enzyme activity of PmCADs expressed as changes in absorbance. **(D)** Reaction product detection with cinnamaldehyde as the substrate using GC-MS. Clac, cinnamyl alcohol. The value is the mean of three biological replicates and SE is shown in the bar chart. ^**^ Represents the significance differences (*p* < 0.01). PmCAD1OE represents a tobacco leaf sample overexpressing *PmCAD1*; PmCAD2OE represents a tobacco leaf sample overexpressing *PmCAD2*; empty vector represents a tobacco leaf sample overexpressing an empty vector.

Furthermore, to verify the role of PmCADs in cinnamyl alcohol synthesis, transiently expressed tobacco leaves combined with enzyme activity analysis were carried out. qRT-PCR was used to detect the expression level of *PmCADs* in tobacco leaves. The results showed that the expression levels of *PmCAD1* and *PmCAD2* in the transiently expressed leaves were much higher than those in the leaves injected with the empty vector (Figures 7A,B). The enzyme activity was measured with cinnamaldehyde as the substrate using a UV spectrophotometer at 340 nm. As shown in Figure 7C, leaves that were overexpressed *PmCAD1* showed a significant catalytic ability, while *PmCAD2* had no significant change compared with the control. Correspondingly, the reaction product was analyzed by GC-MS. It was found that cinnamyl alcohol was detected when overexpressed *PmCAD1* in leaves, while none was detected in that of *PmCAD2* (Figure 7D). The total ion spectra of cinnamaldehyde and cinnamyl alcohol detected are shown in Supplementary Figure 5. In addition, the yield of cinnamyl alcohol was 78.22 mg/g/h (Figure 7D). The results indicated that PmCAD1 could catalyze cinnamaldehyde to cinnamyl alcohol.

### Enzyme Kinetics Analysis of PmCAD1

**FIGURE 8:**
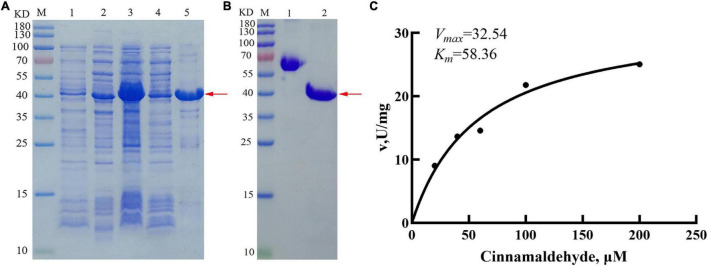
Heterologous expression of PmCAD1 protein in *Escherichia coli*. **(A)** Identification of PmCAD1 with SDS-PAGE analysis; M, protein molecular mass standard; 1, Empty vector; 2, PmCAD1 protein without IPTG; 3, PmCAD1 protein with IPTG; 4, supernatant after induction; and 5, precipitation after induction. **(B)** Protein SDS-PAGE identification. M, protein molecular mass standard; 1, 0.5 mg/ml BSA; and 2, PmCAD1 protein after purification. The red arrow points to PmCAD1 protein. **(C)** Kinetic parameters for purified PmCAD1.

To further analyze the enzyme characterization, recombinant PmCAD1 was induced using 0.2 mM IPTG at 15°C (Figure 8A). After purification, the protein was examined through SDS-PAGE (Figure 8B), In addition, the recombinant protein concentration was 0.6 mg/ml. Kinetic analysis was performed to calculate *K*_*m*_ and *V*_*max*_ values for cinnamaldehyde. As shown in Figure 8C, we obtained a *K*_*m*_ value of 58.36 μM and a *V*_*max*_ value of 32.54 U/mg. This result indicated that PmCAD1 followed the Michaelis-Menten model while catalyzing cinnamaldehyde to cinnamyl alcohol.

## Discussion

It was reported that the floral volatiles of these six cultivars were mainly consisted of phenylpropanoids/benzenoids, including benzyl acetate, eugenol, benzyl alcohol, cinnamyl alcohol, cinnamyl acetate, and benzyl benzoate (Zhang T.X. et al., 2020). According to the previous study, endogenous extraction amounts were basically positively correlated with the emission amounts of benzyl alcohol, benzyl acetate, benzyl benzoate, cinnamyl acetate, and eugenol in each cultivar (Supplementary Figure 6). Especially, the extraction amount and emission amount of benzaldehyde were not consistent. There was no obvious difference in cinnamyl alcohol emission amount between ‘Jia’ and ‘Fenp,’ but the endogenous abundance of cinnamyl alcohol in ‘Fenp’ was more than that in ‘Jia.’ These might be related to the volatilization mechanism that needed us to further explore.

The release of floral fragrance had a temporal rhythm. Farnesol was the main floral compound of *Cymbidium goeringii*. Its release amount showed a trend of rising and then falling and reached the highest emission on the second day after flowering (Ramya et al., 2019). However, the production of essential oils from *Osmanthus fragrans* was the largest at the initial flowering stage and then decreased from the full-blooming stage at the wilting stage (Wang et al., 2009). Cinnamyl alcohol and cinnamyl acetate were the two important floral scent components of ‘Fenp,’ ‘Fenh,’ and ‘Jia.’ There were obvious differences in the release rhythm of cinnamyl alcohol and cinnamyl acetate at different flowering stages. The emission amount of cinnamyl alcohol rises from the budding stage to wilting stage, while the release amount of cinnamyl acetate increased from the budding stage and reached the maximum. In plants, the synthesis of floral volatiles was mainly regulated at the transcriptional level of key enzyme genes in their metabolism (Fan et al., 2015). Combing the expression levels of *PmCAD1* and *PmCAD2* genes (Figure 6) with the emission amount (Figures 1B,C) suggested that PmCAD1 might be involved in the synthesis of cinnamyl alcohol in *P. mume*.

Enzyme activity regulated the volatile synthesis in plants. After adding cinnamaldehyde, each cultivar could catalyze the cinnamaldehyde to synthesize cinnamyl alcohol (Figure 4A), suggesting that the existing substrate cinnamaldehyde might be a limiting factor of cinnamyl alcohol synthesis in ‘Zah,’ ‘Zao,’ and ‘Wuy.’ There were also obvious differences in CAD enzyme activities of six cultivars, which might be regulated by the *PmCAD* transcriptional level. CAD catalyzes cinnamaldehyde into cinnamyl alcohol, providing the substrate for cinnamyl acetate. Furthermore, the phylogenetic evolutionary tree showed that PmCAD1 and PmCAD2 clustered in Class I, among which AtCAD4 and AtCAD5 were considered to be involved in lignin synthesis in *A. thaliana* (Kim et al., 2004). In addition, AaCAD can catalyze aldehydes such as geranial, cinnamaldehyde, artemisinal, and other aldehydes into corresponding alcohols (Li et al., 2012). TaCAD1 also played an important role in the lignification of wheat (Ma, 2010). EgCAD1 could also catalyze a wide of aldehydes, including benzaldehyde, phenylacetaldehyde, cinnamaldehyde, and hydroxycinnamaldehyde (Goffner et al., 1998). These all provide evidence for inferring the function of PmCADs. PmCAD4 was clustered in Class II, in which only PtSAD had been reported to be directly involved in the synthesis of sinapyl alcohol from sinapaldehyde, while the function of other CADs in Class II had not been determined. Thus, different CAD members from the same species might participate in different biosynthesis or have unique catalytic properties.

The amino acid sequences of PmCAD1 and PmCAD2 were conserved. Both contained the βαβ Rossmann fold domain, which was also presented in AtCAD5, SbCAD4, and TaCAD1 (Youn et al., 2006; Sattler et al., 2009; Ma, 2010). In addition, PmCAD1 and PmCAD2 proteins had a highly conserved GLGGVG domain that also was concluded in CADs from other species such as *A. thaliana*, *A. annua*, and *O. sativa* (Tobias and Chow, 2005; Youn et al., 2006; Li et al., 2012). It has been reported that there were 12 residues (i.e., T^49^, Q^53^, L^58^, M^60^, C^95^, W^119^, V^276^, P^286^, M^289^, L^290^, F^299^, and I^300^) participating in the substrate binding and stabilizing cinnamaldehyde aromatic ring (Youn et al., 2006; Tang et al., 2014; Galeano et al., 2018). Nine of out twelve residues provisionally were thought to be conserved and indicative of a character for the CAD family (Youn et al., 2006). Multiple sequence alignment showed that several amino acid residues either in PmCAD1 or PmCAD2 were different from those in AtCAD5, respectively. The residues in PmCAD1 were S^49^, L^95^, and V^300^, and in PmCAD2 were L^95^, T^289^, and V^300^ (Figure 5D). The difference also existed in TaCAD1 and TgCAD1 (Ma, 2010; Galeano et al., 2018). Although I^300^ was considered to be conserved and residues at 49 and 95 showed a conservative heterogeneity among all *bona fide* CAD (Youn et al., 2006), the mutation of S^49^, L^95^, and V^300^ in PmCAD1 and PmCAD2 might not result in the absence of their function. It showed a conservative heterogeneity with Met and Ile for residue 289 (Youn et al., 2006), whereas the residue at 289 in PmCAD2 was threonine, suggesting that it might be the reason why the high sequence similarity but distinct functions of PmCAD1 and PmCAD2 protein, but this hypothesis still needs further analysis.

Most of the CAD proteins reported so far catalyzed cinnamaldehyde to produce cinnamyl alcohol. In this study, its role in the synthesis of cinnamyl alcohol in *P. mume* was verified. Generally, the enzyme activities of CADs in many plants were measured using the prokaryotic expression system, and its characteristics had been analyzed in many plants, such as AaCAD1 from *A. annua* (Li et al., 2012), EgCAD1 from *Eucalyptus gunnii* (Galeano et al., 2018), and MsaCad1 from *M. sativa* (Brill et al., 1999). They could directly catalyze the synthesis of cinnamaldehyde to cinnamyl alcohol. In this study, prokaryotic expression system (Figure 8) and transient expression of *PmCADs* in tobacco leaves (Figure 7) were performed to verify the function. Cinnamyl alcohol was detected in the PmCAD1 reaction product indicating that PmCAD1 could catalyze the synthesis of cinnamyl alcohol. Although the amino acid sequence homology of PmCAD1 and PmCAD2 was as high as 85%, PmCAD1 instead of PmCAD2 had the catalytic activity for cinnamaldehyde. Similarly, the sequence homology between AtCAD4 and AtCAD5 was 83%, and AtCAD5 could catalyze a variety of aldehydes to corresponding alcohols, while AtCAD4 had a low catalytic activity for aldehydes (Kim et al., 2004). However, double mutant (cad-4 cad-5) resulted in a limp floral stem at maturity of *A. thaliana*, suggesting that AtCAD4 and AtCAD5 participated in the formation of coniferyl and sinapyl alcohols in lignifying tissues, and AtCAD4 might have other biological functions (Sibout et al., 2005). In addition, the other function of PmCAD2 in *P. mume* needs to be explored.

## Data Availability Statement

The datasets presented in this study can be found in online repositories. The names of the repository/repositories and accession number(s) can be found below: NCBI PRJNA783623.

## Author Contributions

TZ, FB, and QZ conceived and designed the experiments. TZ and FB prepared the plant materials. TZ performed experiments, analyzed the data, and wrote the manuscript. YY and AD performed the experiments. TZ and QZ played an important role in interpreting the result. TC provided the plant materials. FB and JW revised the manuscript. QZ read and approved the final manuscript. All authors contributed to the article and approved the submitted version.

## Conflict of Interest

The authors declare that the research was conducted in the absence of any commercial or financial relationships that could be construed as a potential conflict of interest.

## Publisher’s Note

All claims expressed in this article are solely those of the authors and do not necessarily represent those of their affiliated organizations, or those of the publisher, the editors and the reviewers. Any product that may be evaluated in this article, or claim that may be made by its manufacturer, is not guaranteed or endorsed by the publisher.

## Footnotes

1
http://sg.idtdna.com/scitools/Applications/RealTimePCR/

2
https://www.omicstudio.cn/tool/59
