# Supplementary material for: Comprehensive Analysis of Endogenous Volatile Compounds, Transcriptome, and Enzyme Activity Reveals PmCAD1 Involved in Cinnamyl Alcohol Synthesis in Prunus mume
Source: Front Plant Sci. 2022 Feb 18;13:820742. doi: 10.3389/fpls.2022.820742 (PMC8894765; doi:10.3389/fpls.2022.820742)
Supplement: Supplementary file 1 [file Data_Sheet_1.docx]

Supplementary Material

Comprehensive analysis of endogenous volatile compounds, transcriptome and enzyme activity reveals *PmCAD1* involved in cinnamyl alcohol synthesis in *Prunus mume*

**Tengxun Zhang^1^, Fei Bao^1^, Aiqin Ding, Yongjuan Yang, Tangren Cheng, Jia Wang, Qixiang Zhang***

Beijing Key Laboratory of Ornamental Plants Germplasm Innovation & Molecular Breeding, National Engineering Research Center for Floriculture, Beijing Laboratory of Urban and Rural Ecological Environment, Engineering Research Center of Landscape Environment of Ministry of Education, Key Laboratory of Genetics and Breeding in Forest Trees and Ornamental Plants of Ministry of Education, School of Landscape Architecture, Beijing Forestry University, Beijing, 100083, China

^1^ These authors contributed equally to this work.

*** Correspondence:**  Qixiang Zhang: zqxbjfu@126.com

**^1^ Co-Authours**

1. **Supplementary Figures and Tables**
   1. **Supplementary Figure**


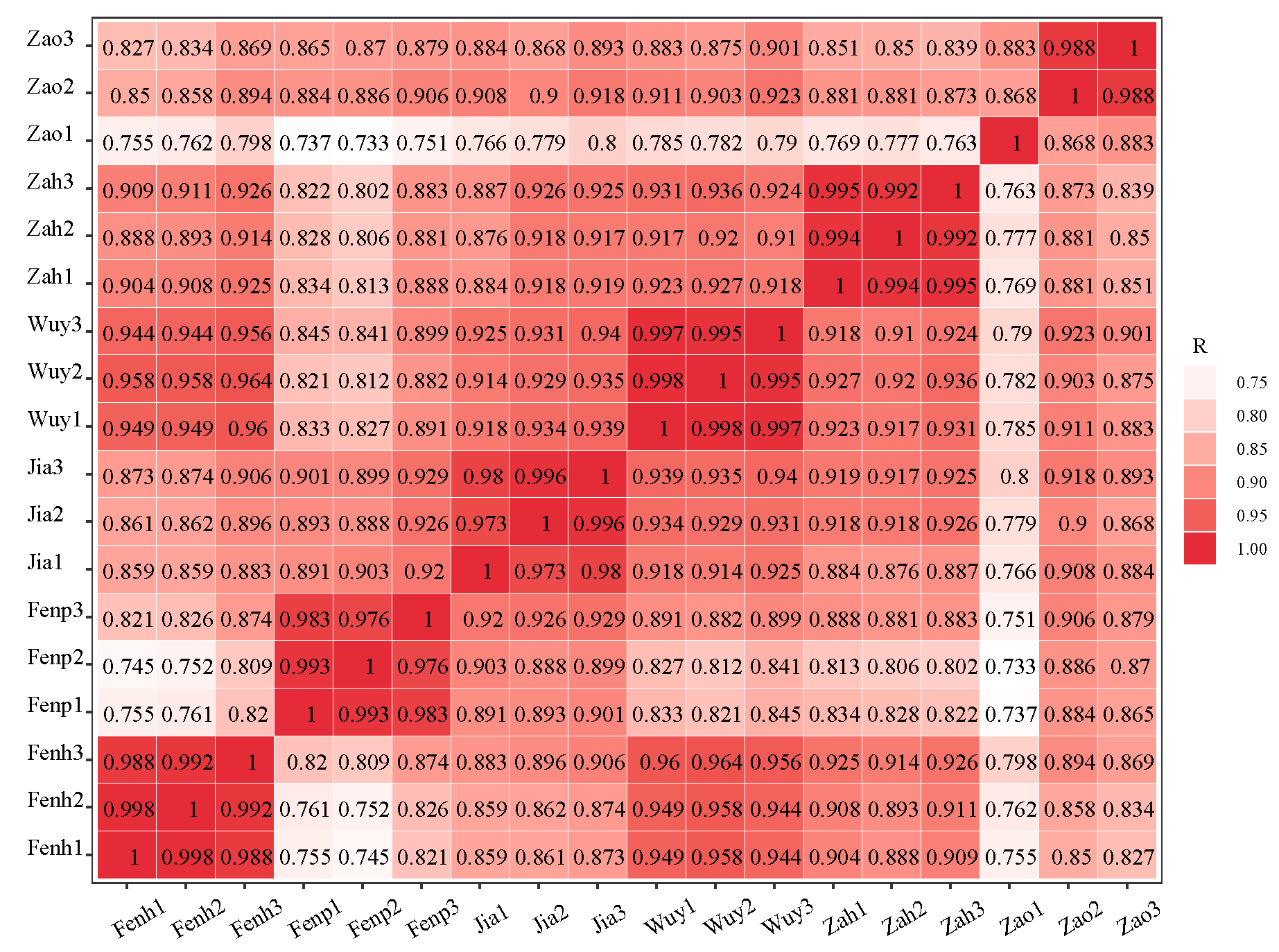


**Supplementary Figure 1.** Correlation analysis of every sample of *P. mume*.


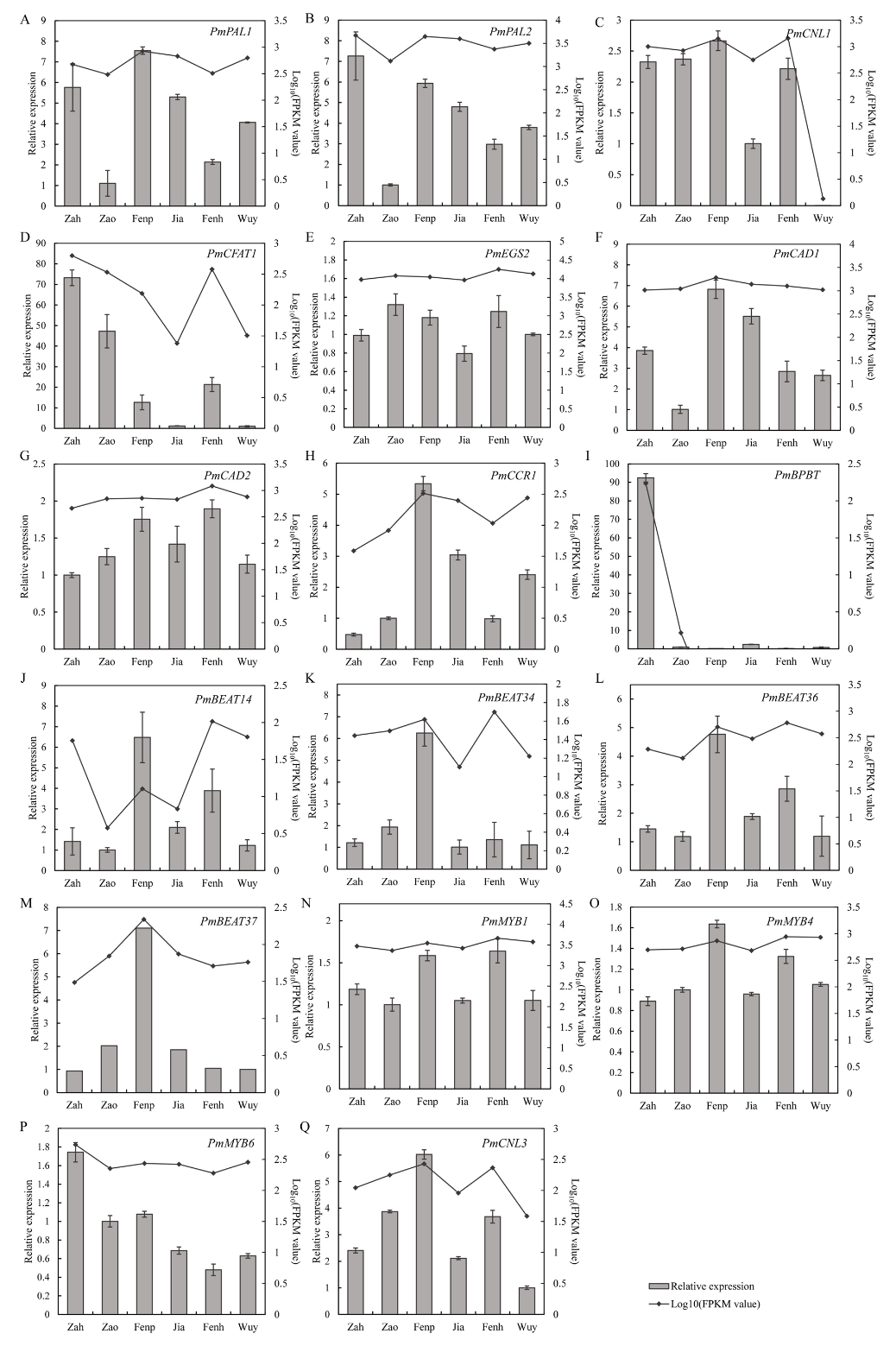


**Supplementary Figure 2.** Expression detection of selected transcripts by RNA-Seq and qRT-PCR. Bar and line chart represent the relative expression level of genes detected by qRT-PCR and transcriptome in flowers at different cultivars, respectively. The value is the mean of three biological replicates and standard error is shown in bar chart. In line chart, the FPKM values were log10 transformed and the dot represents the mean of three values after transformed.


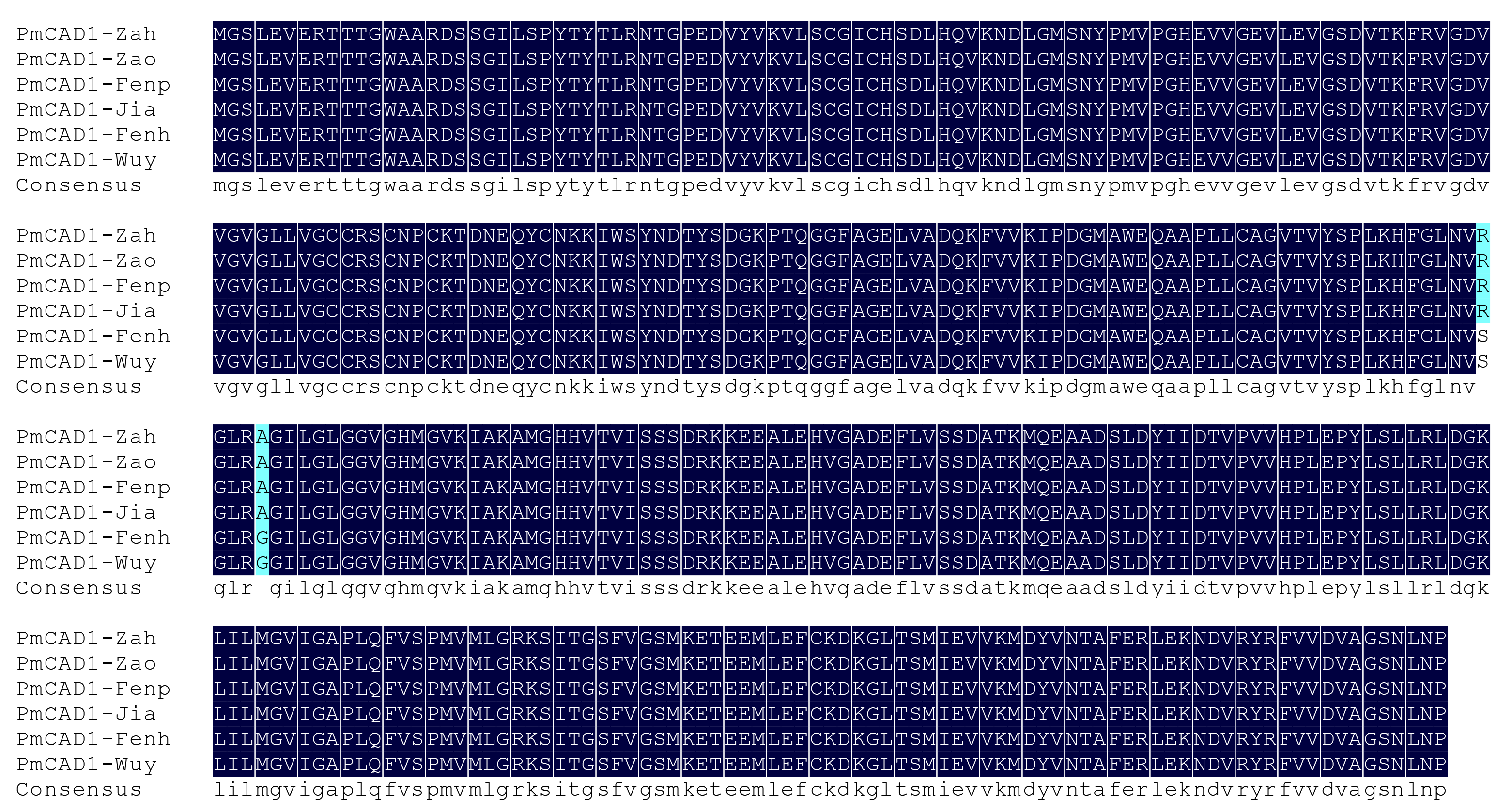


**Supplementary Figure 3.** Multiple sequence alignment of PmCAD1 from six cultivars.


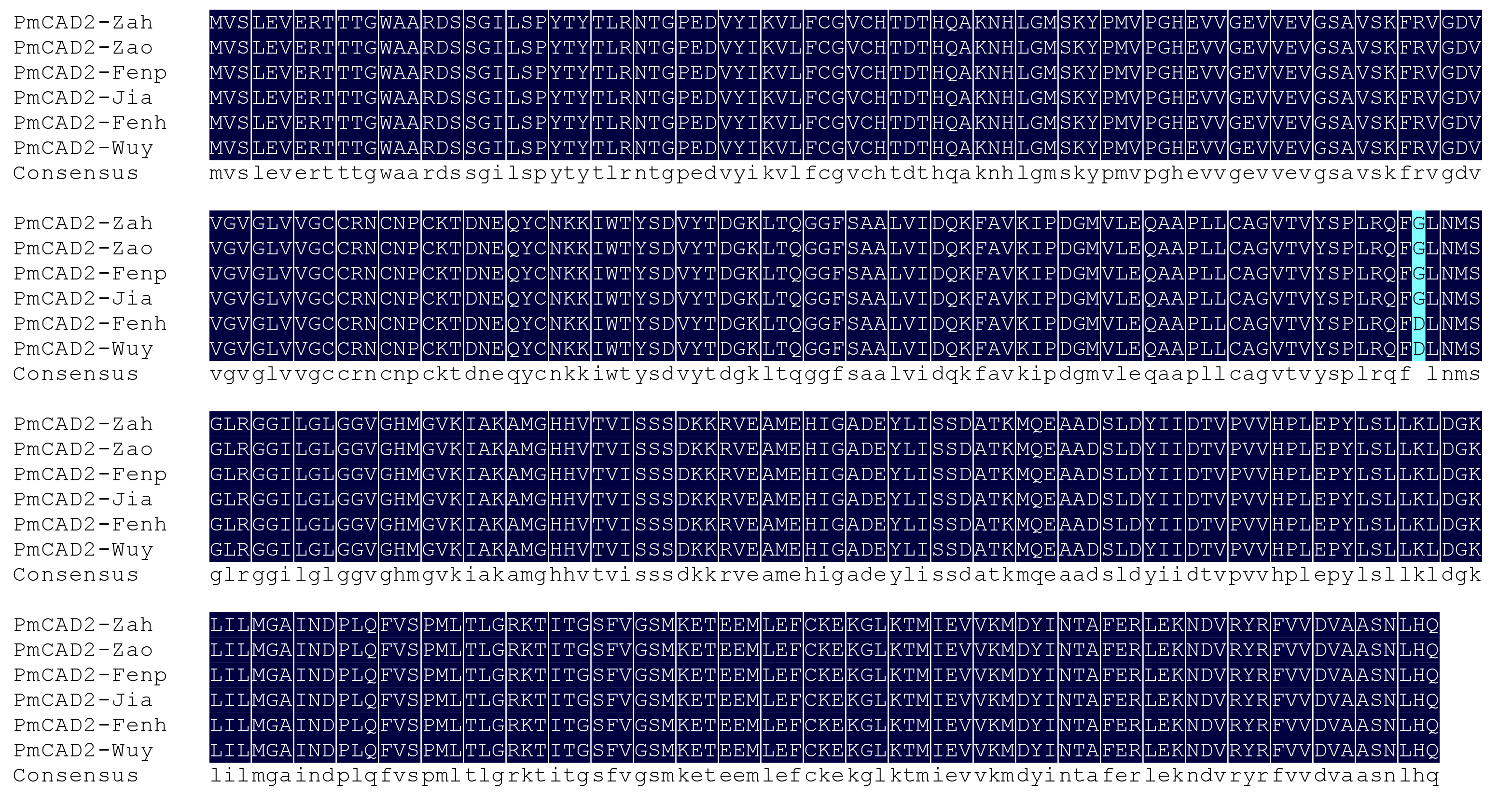


**Supplementary Figure 4.** Multiple sequence alignment of PmCAD2 from six cultivars.


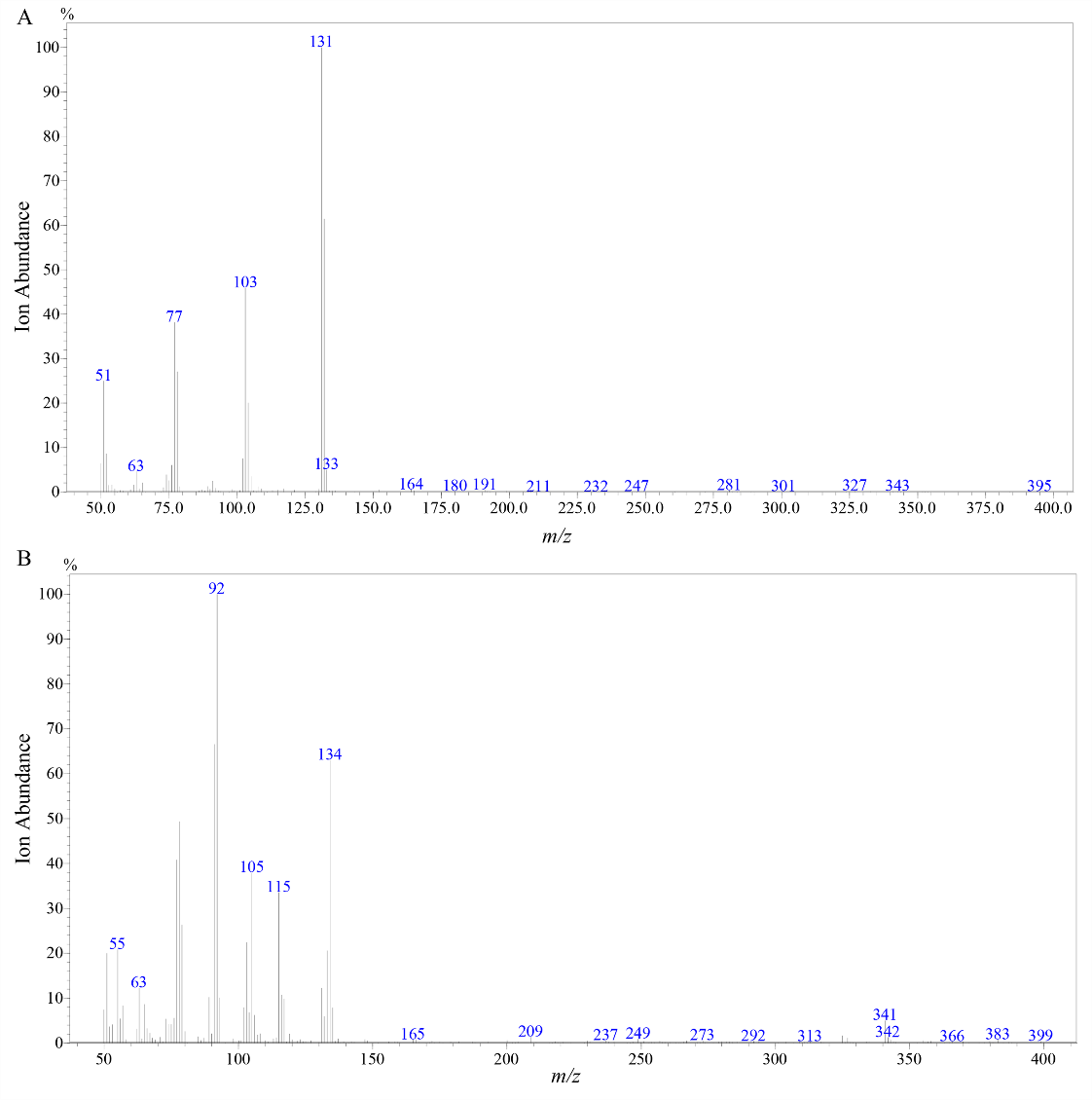


**Supplementary Figure 5.** The mass profiles of cinnamaldehyde and cinnamyl alcohol. (A): cinnamaldehyde; (B): cinnamyl alcohol.


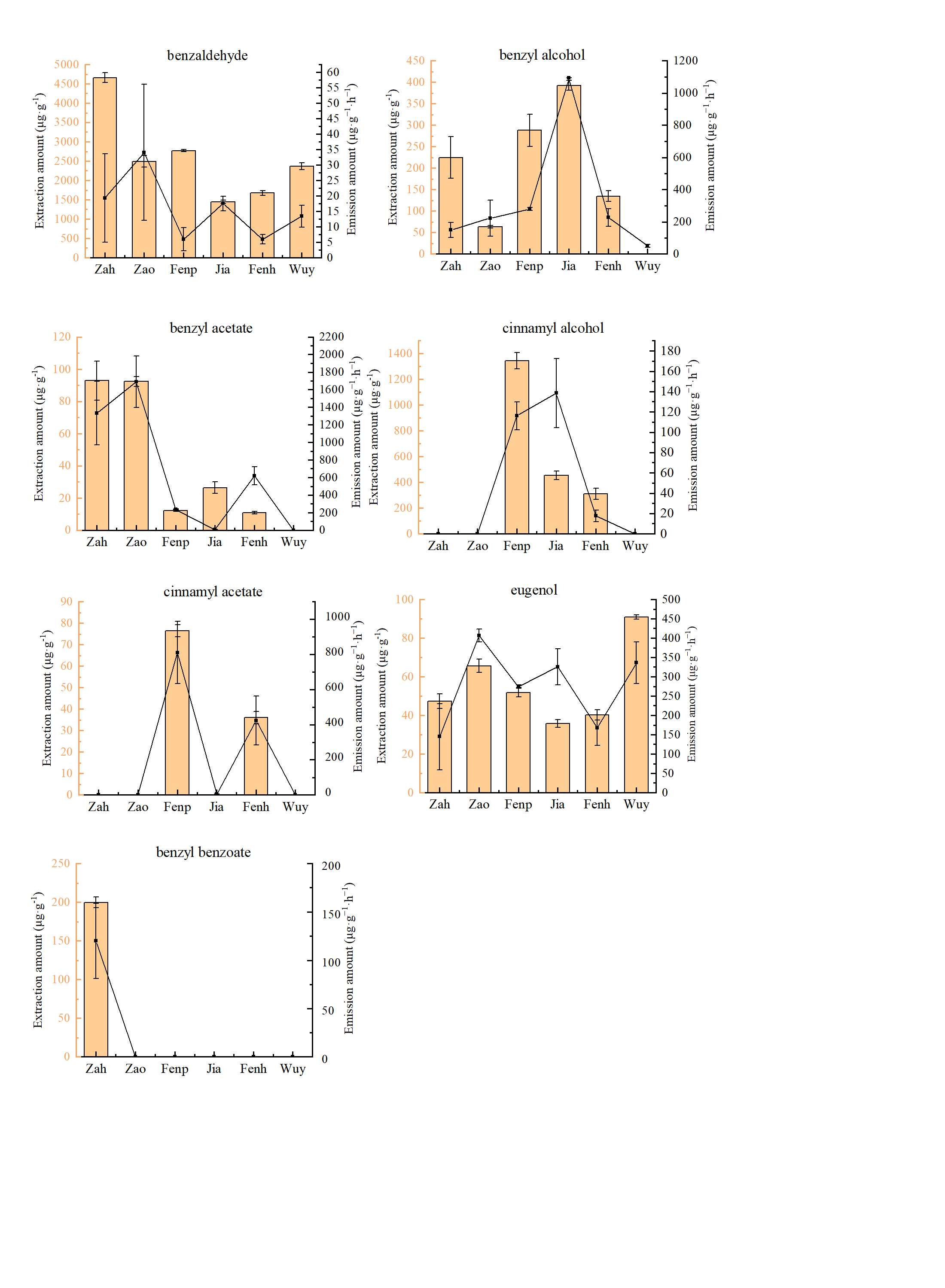


**Supplementary Figure 6.** Extraction and emission amount of main floral scent compounds in the six *P. mume* cultivars. The orange bar graph represents the extraction amount and the black line graph represents the emission amount. The data was the average value of three replicates.

## Supplementary Tables

**Supplementary Table 1.** Primer sequences of candidate genes for real-time PCR.

| Primer name | Gene name | Primer sequence (5'→3') |
| --- | --- | --- |
| *Pm012986* | *PmCNL1-qF* | AATGAACATGCTTCCCAAATG |
|  | *PmCNL1-qR* | CTGAGGTTCGGTTAGCATAAA |
| *Pm012962* | *PmCNL3-qF* | CCAAACTCATCTCCTCTTACTC |
|  | *PmCNL3-qR* | GTGTAGGTGGTATCGTTGTAAA |
| *Pm030127* | *PmPAL2-qF* | TGGGTGCAGAGTACCTAACA |
|  | *PmPAL2-qR* | CAATCCAGAATCGGGTCGATAA |
| *Pm018524* | *PmPAL1-qF* | GGGTTGATGGTGGGTTCTT |
|  | *PmPAL1-qR* | GGCCTCAAAGAGAACCATAGAA |
| *Pm017753* | *PmBPBT-qF* | TGGCAGATCTCATGGTTATCAG |
|  | *PmBPBT-qR* | CCAAAGTCCACCTCTCCAAA |
| *Pm010967* | *PmBEAT14-qF* | CTATTCCTCGAGAACCA |
|  | *PmBEAT14-qR* | GTGAGAGTTTTAGCTAATGACC |
| *Pm011001* | *PmBEAT34-qF* | GTCTACGGAAAGTGAAGTG |
|  | *PmBEAT34-qR* | CAATGATAGGTCTCAGTGTTA |
| *Pm011009* | *PmBEAT36-qF* | CGAGTATGGAGGAATAGCAA |
|  | *PmBEAT36-qR* | TCATCGTCACCAACAGAA |
| *Pm011010* | *PmBEAT37-qF* | TGGTGACGGTGTTGCCAC |
|  | *PmBEAT37-qR* | ATCATCGTGACAATCATCAAG |
| *Pm021215* | *PmCAD1-qF* | TCGCTGGTGAACTGGTCGCT |
|  | *PmCAD1-qR* | TCCTGCTCTTAATCCTCTCA |
| *Pm021214* | *PmCAD2-qF* | TTTCTGCTGCCCTAGTCATC |
|  | *PmCAD2-qR* | GCCTCCTCTTAACCCACTCAT |
| *Pm012335* | *PmCCR1-qF* | TGTGCGAAATCCCAGTCTATC |
|  | *PmCCR1-qR* | ACCAAGTCTCATCAACAACTACA |
| *Pm006362* | *PmPP2A-qF* | ATATAGCTGCTCAGTTCAACC |
|  | *PmPP2A-qR* | AAAAACAGTCACCACATTCTT |
| *Pm013138* | *PmCFAT1-qF* | CTAAATCTGACACTAAAATTGGTT |
|  | *PmCFAT1-qR* | TCAACGACTTTTCAACTAGGTCTTCC |
| *Pm012360* | *PmEGS2-qF* | TCTACGCTGCCTATCCCTCAAC |
|  | *PmEGS2-qR* | AGCCCTTCTAATCTTCCTTCTATC |
| *Pm015692* | *PmMYB1-qF* | TGCTGTGGACACAATGGAA |
|  | *PmMYB1-qR* | GGGAGTGATCAGCAGGTAAAG |
| *Pm020852* | *PmMYB4-qF* | GCTTCTGGGCAAAGACTCA |
|  | *PmMYB4-qR* | AGGATCACTGGGTTGTTGTG |
| *Pm021211* | *PmMYB6-qF* | GATCTCGGGAAACGACGATG |
|  | *PmMYB6-qR* | GCTGTAACAGAGGCCAGAAA |

**Supplementary Table 2.** Accession number of CAD sequences used in the phylogenetic tree.

| Name | Species | Accession number |
| --- | --- | --- |
| PmCAD1 | *P. mume* | LOC103335153 |
| PmCAD2 | *P. mume* | LOC103335152 |
| PmCAD3 | *P. mume* | LOC103329119 |
| PmCAD4 | *P. mume* | LOC103335277 |
| AcCAD1 | *Aralia cordata* | BAA03099.1 |
| AaCAD1 | *Artemisia annua* | ACB54931.1 |
| AtCAD2 | *Arabidopsis thaliana* | AAP59430.1 |
| AtCAD3 | *A. thaliana* | AAP59431.1 |
| AtCAD4 | *A. thaliana* | AAP59434.1 |
| AtCAD5 | *A. thaliana* | AAP59435.1 |
| AtCAD6 | *A. thaliana* | AAP59428.1 |
| AtCAD7 | *A. thaliana* | AAP59432.1 |
| AtCAD8 | *A. thaliana* | AAP59433.1 |
| AtCAD9 | *A. thaliana* | AAP59429.1 |
| EgCAD1 | *Eucalyptus globulus* | AAC07987.1 |
| MsaCAD1 | *Medicago sativa* | AAC35846.1 |
| MsaCAD2 | *M. sativa* | AAC35845.1 |
| MtCAD1 | *Medicago truncatula* | KEH44099.1 |
| NtCAD1 | *Nicotiana tabacum* | P30359.1 |
| NtCAD2 | *N. tabacum* | P30360 |
| OsCAD1 | *Oryza sativa* | AAN09864.1 |
| OsCAD2 | *O. sativa* | ABB04029.1 |
| OsCAD7 | *O. sativa* | CAE05206.3 |
| OsCAD9 | *O. sativa* | AAN05338.1 |
| PtCAD1 | *Populus tremuloides* | AAF43140.1 |
| PtSAD | *P. tremuloides* | AAK58693.1 |
| TaCAD1 | *Triticum aestivum* | ADI59734.1 |
| TgCAD1 | *Tectona grandis* | ANG60951.1 |
| TgCAD2 | *T. grandis* | ANG60952.1 |
| TgCAD3 | *T. grandis* | ANG60953.1 |
| TgCAD4 | *T. grandis* | ANG60954.1 |
| ZmCAD1 | *Zea mays* | O24562 |
| CmCAD2 | *Cucumis melo var. makuwa* | TYK06792.1 |
| StDRD1 | *Solanum tuberosum* | CAD29291.1. |

**Supplementary Table 3.** Primer sequences of candidate genes for gene clone and vector construction.

| Gene name | Primer sequence (5'→3') | Usage |
| --- | --- | --- |
| *PmCAD1-F* | ATGGGTAGCCTTGAAGTGGAGAGAA | Gene clone |
| *PmCAD1-R* | CTATGGATTAAGATTGCTGCCGGC |  |
| *PmCAD2-F* | ATGGTTAGCCTTGAGGTGGAGA |  |
| *PmCAD2-R* | CTATTGATGAAGATTGCTGGCAG |  |
| *PmCAD1-vF* | CCAAATCGACTCTAGAATGGGTAGCCTTGAAGTGGAGAGAA | Plant expression vector construction |
| *PmCAD1-vR* | CCCTTGCTCACCATGGTACCTGGATTAAGATTGCTGCCGGC |  |
| *PmCAD2-vF* | CCAAATCGACTCTAGAATGGTTAGCCTTGAGGTGGAGA |  |
| *PmCAD2-vR* | CCCTTGCTCACCATGGTACCTTGATGAAGATTGCTGGCAG |  |
| *NtEFα1-qf* | TGGTTGTGACTTTTGGTCCCA | qRT-PCR |
| *NtEFα1-qr* | ACAAACCCACGCTTGAGATCC |  |
| *PmCAD1-vF1* | GGAGATATACATATGATGGGTAGCCTTGAAGTGGAGAGAA | Prokaryotic expression vector construction |
| *PmCAD1-vR1* | GTGGTGGTGCTCGAGTGGATTAAGATTGCTGCCGGC |  |

**Supplementary Table 4.** Summary of the total RNA quality testing.

| Samples | Raw Data | | Valid Data | | Valid Ratio（reads） | Q20% | Q30% | GC content% |
| --- | --- | --- | --- | --- | --- | --- | --- | --- |
|  | Read | Base (G) | Read | Base  (G) |  |  |  |  |
| Zah1 | 44313480 | 6.65 | 43496958 | 6.52 | 98.16 | 99.97 | 97.85 | 46 |
| Zah2 | 45194598 | 6.78 | 44310560 | 6.65 | 98.04 | 99.98 | 97.88 | 47 |
| Zah3 | 51264502 | 7.69 | 50512030 | 7.58 | 98.53 | 99.98 | 98.55 | 46 |
| Zao1 | 53238168 | 7.99 | 52060556 | 7.81 | 97.79 | 99.98 | 97.9 | 46 |
| Zao2 | 42476510 | 6.37 | 40900532 | 6.14 | 96.29 | 99.99 | 98.11 | 45.5 |
| Zao3 | 43297720 | 6.49 | 42554718 | 6.38 | 98.28 | 99.98 | 97.9 | 46 |
| Fenp1 | 48340894 | 7.25 | 47765656 | 7.16 | 98.81 | 99.98 | 98.55 | 46 |
| Fenp2 | 48029210 | 7.2 | 47488896 | 7.12 | 98.88 | 99.98 | 98.65 | 46 |
| Fenp3 | 49170348 | 7.38 | 48550078 | 7.28 | 98.74 | 99.98 | 98.51 | 46 |
| Jia1 | 44207462 | 6.63 | 43578452 | 6.54 | 98.58 | 99.99 | 98.56 | 46 |
| Jia2 | 48388680 | 7.26 | 47743330 | 7.16 | 98.67 | 99.99 | 98.42 | 46 |
| Jia3 | 43241602 | 6.49 | 42687564 | 6.4 | 98.72 | 99.99 | 98.62 | 46 |
| Fenh1 | 42087500 | 6.31 | 41515918 | 6.23 | 98.64 | 99.98 | 98.5 | 46 |
| Fenh2 | 45167158 | 6.78 | 44365048 | 6.65 | 98.22 | 99.98 | 97.76 | 45.5 |
| Fenh3 | 47023618 | 7.05 | 46341670 | 6.95 | 98.55 | 99.97 | 97.83 | 45.5 |
| Wuy1 | 44758318 | 6.71 | 44037380 | 6.61 | 98.39 | 99.97 | 97.72 | 45.5 |
| Wuy2 | 50140344 | 7.52 | 49469362 | 7.42 | 98.66 | 99.98 | 97.81 | 46 |
| Wuy3 | 49706792 | 7.46 | 48997482 | 7.35 | 98.57 | 99.98 | 97.77 | 45.5 |

**Supplementary Table 5.** Statistics of reads compared with mei’s genome

| Samples | Valid reads | Mapped reads (%) | PE Mapped reads (%) | Non-splice reads (%) | Splice reads (%) |
| --- | --- | --- | --- | --- | --- |
| Zah1 | 43496958 | 93.46 | 85.15 | 58.48 | 31.34 |
| Zah2 | 44310560 | 91.64 | 82.51 | 60.28 | 27.5 |
| Zah3 | 50512030 | 93.35 | 83.87 | 58.6 | 31.23 |
| Zao1 | 52060556 | 91.43 | 81.71 | 58.51 | 29.75 |
| Zao2 | 40900532 | 90.81 | 76.45 | 59.09 | 28.92 |
| Zao3 | 42554718 | 91.47 | 84.07 | 57.63 | 30.88 |
| Fenp1 | 47765656 | 91.39 | 82.64 | 56.92 | 31.4 |
| Fenp2 | 47488896 | 91.09 | 82.41 | 56.88 | 31.03 |
| Fenp3 | 48550078 | 90.76 | 80.84 | 57.15 | 30.5 |
| Jia1 | 43578452 | 93.88 | 84.15 | 59.4 | 31.16 |
| Jia2 | 47743330 | 93.68 | 84.22 | 58.51 | 31.93 |
| Jia3 | 42687564 | 93.86 | 84.94 | 58.87 | 31.81 |
| Fenh1 | 41515918 | 93.27 | 83.55 | 58.86 | 31.25 |
| Fenh2 | 44365048 | 92.68 | 81.94 | 58.98 | 30.5 |
| Fenh3 | 46341670 | 92.92 | 83.45 | 58.47 | 31.22 |
| Wuy1 | 44037380 | 89.49 | 80.89 | 56.83 | 29.69 |
| Wuy2 | 49469362 | 89.48 | 81.17 | 57.23 | 29.22 |
| Wuy3 | 48997482 | 89.47 | 80.89 | 57.09 | 29.41 |

**Supplementary Table 6.** FPKM values of the key genes that were differentially expressed among the six cultivars of *P. mume*.

| **Name** | **Gene ID** | **Zah-FPKM** | **Zao-FPKM** | **Fenp-FPKM** | **Jia-**  **FPKM** | **Fenh-FPKM** | **Wuy-FPKM** | **p-value** | **significant** |
| --- | --- | --- | --- | --- | --- | --- | --- | --- | --- |
| PAL | Pm018524 | 471.68 | 304.88 | 839.41 | 676.00 | 320.77 | 628.84 | 0.00 | yes |
| PAL | Pm030127 | 4703.07 | 1311.28 | 4463.42 | 3967.99 | 2370.93 | 3169.44 | 0.00 | yes |
| C4H | Pm000242 | 484.81 | 344.67 | 389.66 | 347.93 | 287.80 | 395.03 | 0.01 | yes |
| 4CL | Pm008736 | 618.24 | 456.98 | 660.01 | 675.99 | 832.05 | 938.75 | 0.00 | yes |
| 4CL | Pm013887 | 6.60 | 7.23 | 5.21 | 4.07 | 3.16 | 7.87 | 0.00 | yes |
| 4CL | Pm019600 | 51.98 | 36.37 | 36.12 | 36.19 | 17.88 | 49.26 | 0.00 | yes |
| CNL | Pm012986 | 1003.57 | 844.14 | 1415.38 | 560.55 | 1441.82 | 1.35 | 0.00 | yes |
| CCR | Pm000200 | 6.43 | 11.34 | 6.63 | 9.04 | 4.90 | 9.23 | 0.00 | yes |
| CCR | Pm000201 | 2.43 | 5.89 | 1.87 | 3.70 | 2.01 | 3.98 | 0.00 | yes |
| CCR | Pm000202 | 11.50 | 15.30 | 10.35 | 17.02 | 9.36 | 15.95 | 0.00 | yes |
| CCR | Pm003802 | 2.98 | 3.13 | 4.02 | 1.56 | 2.08 | 1.32 | 0.00 | yes |
| CCR | Pm008602 | 26.17 | 56.88 | 13.10 | 45.58 | 10.31 | 46.74 | 0.00 | yes |
| CCR | Pm012335 | 38.54 | 82.15 | 325.15 | 249.12 | 107.21 | 276.04 | 0.00 | yes |
| CCR | Pm013776 | 2250.15 | 1135.24 | 3852.79 | 1623.13 | 3299.16 | 2669.50 | 0.00 | yes |
| CCR | Pm013782 | 3154.72 | 1251.15 | 3000.76 | 206.06 | 1975.96 | 304.28 | 0.00 | yes |
| CCR | Pm013786 | 35.86 | 58.16 | 15.61 | 18.02 | 27.47 | 28.28 | 0.00 | yes |
| CCR | Pm022900 | 7.89 | 9.88 | 7.63 | 6.89 | 9.69 | 12.02 | 0.01 | yes |
| CCR | Pm022901 | 49.86 | 74.11 | 33.71 | 47.93 | 47.83 | 91.22 | 0.00 | yes |
| CCR | Pm022902 | 2.95 | 3.55 | 28.01 | 2.02 | 0.76 | 1.30 | 0.00 | yes |
| CCR | Pm023202 | 391.85 | 201.78 | 371.61 | 453.79 | 155.66 | 210.81 | 0.00 | yes |
| PmCAD5 | Pm001694 | 27.09 | 11.13 | 8.17 | 8.95 | 12.13 | 1.71 | 0.00 | yes |
| PmCAD4 | Pm002261 | 3.48 | 6.75 | 105.89 | 79.62 | 13.38 | 39.40 | 0.00 | yes |
| PmCAD6 | Pm006164 | 11.07 | 2.77 | 1.28 | 5.53 | 13.90 | 0.97 | 0.00 | yes |
| PmCAD3 | Pm014932 | 1.64 | 5.15 | 146.83 | 207.09 | 28.62 | 23.52 | 0.00 | yes |
| PmCAD2 | Pm021214 | 460.80 | 698.16 | 717.33 | 677.68 | 1211.86 | 759.60 | 0.00 | yes |
| PmCAD1 | Pm021215 | 1033.00 | 1097.00 | 1916.67 | 1379.60 | 1264.43 | 1048.11 | 0.00 | yes |
| PmCAD7 | Pm024279 | 11.53 | 25.39 | 2.14 | 5.38 | 17.83 | 6.03 | 0.00 | yes |
| BAHD | Pm006860 | 13.91 | 15.98 | 8.55 | 10.41 | 14.58 | 9.01 | 0.00 | yes |
| BAHD | Pm006894 | 7.00 | 4.14 | 3.10 | 3.02 | 7.29 | 4.84 | 0.00 | yes |
| BAHD | Pm010967 | 57.19 | 3.77 | 12.72 | 6.83 | 103.95 | 63.87 | 0.00 | yes |
| BAHD | Pm011001 | 27.82 | 31.28 | 41.56 | 12.74 | 49.90 | 16.60 | 0.00 | yes |
| BAHD | Pm011005 | 18.75 | 21.75 | 11.93 | 18.28 | 30.59 | 6.08 | 0.00 | yes |
| BAHD | Pm011009 | 193.32 | 130.68 | 505.71 | 304.07 | 607.01 | 374.18 | 0.00 | yes |
| BAHD | Pm011010 | 30.74 | 69.75 | 218.34 | 74.33 | 51.12 | 57.63 | 0.00 | yes |
| BAHD | Pm011011 | 11.61 | 40.16 | 16.42 | 15.69 | 7.65 | 22.27 | 0.00 | yes |
| BAHD | Pm011034 | 20.92 | 102.97 | 5.93 | 2.63 | 16.29 | 1.92 | 0.00 | yes |
| BAHD | Pm026103 | 1.09 | 1.93 | 2.84 | 0.87 | 1.72 | 0.84 | 0.00 | yes |
| BAHD | Pm028798 | 5.56 | 16.24 | 5.29 | 3.04 | 1.64 | 6.27 | 0.00 | yes |
| BAHD | Pm016771 | 3.24 | 1.88 | 0.36 | 0.23 | 3.94 | 2.98 | 0.00 | yes |
| CFAT | Pm013138 | 631.57 | 339.70 | 154.13 | 23.99 | 379.82 | 32.07 | 0.00 | yes |
| CFAT | Pm030672 | 353.19 | 179.00 | 71.83 | 12.59 | 268.37 | 20.15 | 0.00 | yes |
| CFAT | Pm030674 | 66.46 | 55.31 | 12.82 | 1.99 | 36.59 | 2.45 | 0.00 | yes |
| BPBT | Pm017753 | 171.36 | 1.65 | 0.12 | 0.23 | 0.18 | 0.28 | 0.00 | yes |
| EGS | Pm012358 | 240.07 | 688.10 | 115.37 | 347.71 | 796.02 | 936.15 | 0.00 | yes |
| EGS | Pm012355 | 220.62 | 626.70 | 299.34 | 321.86 | 677.01 | 817.20 | 0.00 | yes |
| EGS | Pm012360 | 9362.31 | 11829.50 | 11008.26 | 9104.56 | 17682.85 | 13376.39 | 0.00 | yes |
